# Small RNA Modules Confer Different Stabilities and Interact Differently with Multiple Targets

**DOI:** 10.1371/journal.pone.0052866

**Published:** 2013-01-22

**Authors:** José Marques Andrade, Vânia Pobre, Cecília Maria Arraiano

**Affiliations:** Instituto de Tecnologia Química e Biológica, Universidade Nova de Lisboa Av. da República, Oeiras, Portugal; The Methodist Hospital Research Institute, United States of America

## Abstract

Bacterial Hfq-associated small regulatory RNAs (sRNAs) parallel animal microRNAs in their ability to control multiple target mRNAs. The small non-coding MicA RNA represses the expression of several genes, including major outer membrane proteins such as *omp*A, *tsx* and *ecn*B. In this study, we have characterised the RNA determinants involved in the stability of MicA and analysed how they influence the expression of its targets. Site-directed mutagenesis was used to construct MicA mutated forms. The 5′linear domain, the structured region with two stem-loops, the A/U-rich sequence or the 3′ poly(U) tail were altered without affecting the overall secondary structure of MicA. The stability and the target regulation abilities of the wild-type and the different mutated forms of MicA were then compared. The 5′ domain impacted MicA stability through an RNase III-mediated pathway. The two stem-loops showed different roles and disruption of stem-loop 2 was the one that mostly affected MicA stability and abundance. Moreover, STEM2 was found to be more important for the *in vivo* repression of both *omp*A and *ecn*B mRNAs while STEM1 was critical for regulation of *tsx* mRNA levels. The A/U-rich linear sequence is not the only Hfq-binding site present in MicA and the 3′ poly(U) sequence was critical for sRNA stability. PNPase was shown to be an important exoribonuclease involved in sRNA degradation. In addition to the 5′ domain of MicA, the stem-loops and the 3′ poly(U) tail are also shown to affect target-binding. Disruption of the 3′U-rich sequence greatly affects all targets analysed. In conclusion, our results have shown that it is important to understand the “sRNA anatomy” in order to modulate its stability. Furthermore, we have demonstrated that MicA RNA can use different modules to regulate its targets. This knowledge can allow for the engineering of non-coding RNAs that interact differently with multiple targets.

## Introduction

Small RNA-mediated networks control a wide variety of cellular processes. The development of new experimental strategies has contributed enormously to the increasing number of small RNAs identified in bacteria [1]. About 100 small RNAs have been experimentally confirmed in *Escherichia coli* and many more have been predicted [2]. Comparative profiling of strains has contributed to the identification of novel non-coding RNAs in other bacteria [3]. Small RNAs are distinct amongst themselves and their structural diversity makes it difficult to unify this class of cell regulators. sRNAs are diverse in size and do not display a common sequence that can be used as a signature [4]. They present diverse modes of action, exerting either a positive or a negative effect on the expression of the target mRNAs.

The interactions between sRNA and mRNAs contribute to the differential modulation of gene expression. Bacterial *trans*-encoded small RNAs bind to their target mRNAs through the establishment of short and imperfect antisense base pairing interactions in a close parallel to the action of eukaryotic miRNAs [5]. The base pairing of sRNAs can take place at different sites on the target but they usually occur within the 5′ end of the mRNA [6]–[9]. It was shown that sRNA-mRNA pairs can be subject to endonucleolytic degradation, in which RNase E and RNase III play major roles [10], [11]. However, as result of the base pairing dynamics not all the population of a small RNA is going to be bound to its targets. Degradation of the free fraction may unbalance the pool of available small RNAs. The 3′-5′ exoribonucleolytic degradation plays an important role in this regulation and PNPase was shown to be a major enzyme in the small RNAs turnover [12], [13]. Therefore, the study of the elements controlling the sRNA stability is critical to better understand the regulation of the sRNA-based pathways.

**Figure 1:**
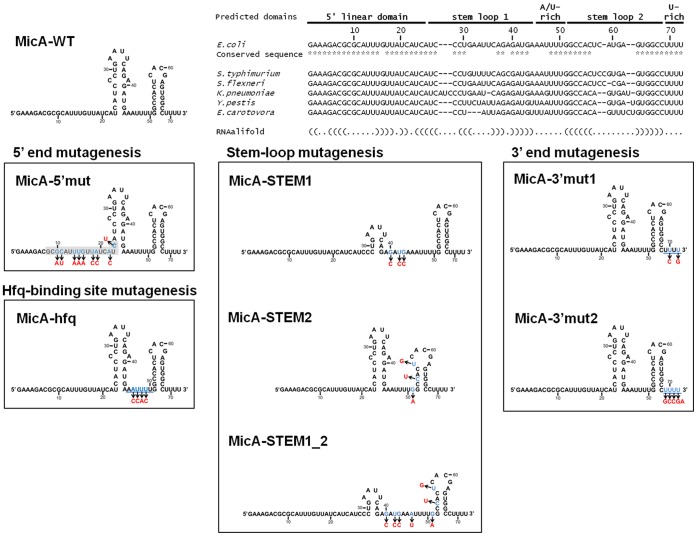
Construction of MicA mutants. Nucleotide sequence and secondary structure of the *E. coli* wild-type MicA is shown on top. The proposed modular domain organization of MicA is indicated. MicA sequence and structure is conserved in several enterobacteria as analysed by the CLUSTALW [65] and the RNAalifold software [66]. The model structures predicted with mfold [18] for the different MicA mutants are schematized. Mutagenesis of the different domains was designed to not disturb the overall secondary structure of the MicA RNA. Nucleotides changes are indicated by arrows.

The well characterised small RNA MicA was used as model for our study. MicA was initially identified to repress the synthesis of several major outer membrane proteins (OMPs) [14]–[16]. The list of target mRNAs for MicA was recently expanded through the use of microarray studies [17]. MicA belongs thus to the increasing cluster of sRNAs that regulate multiple targets. The architecture of a small RNA greatly contributes to its stability and may define its ability to interact with different target mRNAs. Enzymatic and chemical probing was used to map the structure of MicA which was essentially consistent with the conformation predicted by the mfold algorithm [15], [18]. Based on the sequence and secondary structure, we have defined the following domains in MicA: a 5′ linear sequence; a Hfq-binding A/U-rich sequence; two structured elements (stem-loops) and finally a U-rich linear stretch in the 3′ end (Figure 1 and Figure S1). A similar modular structure was proposed to other small RNAs, like RybB and SgrS [19]–[22].

The 5′ end of MicA was suggested to be the principal target recognition domain, like it happens for many other small RNAs [23]. For the RybB RNA it was even possible to define a short “seed” sequence located in the 5′ end that is responsible for interaction with multiple targets in *Salmonella* [19], [21]. However, this does not seem to be the common rule and the nucleotides and the length of the 5′ sRNA sequence involved in the regulation of the different targets may differ [7].

All the *E. coli trans*-encoded sRNA such as MicA bind to RNA chaperone Hfq, a protein homologous to Sm and Sm-like proteins involved in RNA processing in eukaryotes [24], [25]. Hfq plays multiple roles in the cell but mostly facilitates the sRNA-mRNA annealing [26], [27]. It was found to bind linear A/U-rich sequences in RNA and was recently shown to preferably interact with the 3′ end of small RNAs [28]–[30]. Hfq is also very important for sRNA stabilization and was shown to protect from RNase E- and PNPase-mediated degradation [13], [31].

Although sRNAs are usually structured molecules, the degree of RNA folding varies according to the GC-content of the sRNA analysed. A stem-loop corresponding to the *rho*-independent transcriptional terminator followed by a short U-rich sequence is ubiquitous amongst small RNAs [32], [33]. Very recently, Hfq was shown to bind to this sequence which makes this a potential domain for interaction with mRNA [20], [28], [30]. The presence of additional stems is frequent in many sRNAs and MicA harbours a total of two stem-loops. These structures can potentially act as stabilizer elements as they can hinder the 3′-5′ exonucleolytic degradation pathway of many RNA substrates [34]. The structure of the sRNA can also be critical for interaction with the target mRNA and conformational rearrangements can lead to disruption of sRNA-mRNA base pairing. This is well illustrated in many studies such as the OxyS interaction with the *fhlA* mRNA [35], [36] or the base pairing between RyhB and the *isc*RSUA policistronic transcript [37].

Through use of mutational studies we analysed the modular domain organization of MicA. Several nucleotide changes were introduced in the *mic*A gene in order to disrupt independently each predefined domain without affecting the overall conformation of the molecule. We demonstrate that several elements present in the 3′ end of MicA act as stabilizers of this small RNA and this region is also suggested to play important roles in the MicA-dependent riboregulation of different target mRNAs. In addition to the well characterised role of the 5′ end of MicA in the interaction with targets we suggest that elements present in the 3′ end of MicA can also contribute to the differential regulation of the mRNA targets.

## Results

### 1. Engineering Synthetic MicA RNAs

Base pairing with target mRNAs is determinant for small RNA function and stability. The 5′ region of MicA was predicted to be the major region involved in the interaction with the target mRNAs. However, additional elements present in the small RNA molecule can potentially influence MicA activity and/or stability. From the analysis of MicA sequence and its secondary structure (as reported in [15]) we have defined the following small RNA modules: a 5′-end linear stretch, a structured region harbouring two stem-loops (STEM1 and STEM2) separated by an A/U-rich sequence and finally the transcriptional termination U-rich sequence located at the 3′-end (Figure 1). The 5′ end and the far most 3′ end nucleotides of MicA are highly conserved while the regions encompassing the stem-loops can comprise some variability amongst enterobacteria, as observed in the sequence alignment in Figure 1.

In order to get insights into the relationship between the architecture, the function and stability of a small RNA, we constructed several altered MicA’s using the technique of overlapping PCR. The nucleotide changes introduced are easily identified in the schematic representation of MicA variants (Figure 1 and Figure S1). RNA secondary structure might be critical for the function of a small RNA. The chemical mapping of the structure of MicA RNA was previously performed and essentially agreed with the mfold algorithm analysis [15], [18]. Accordingly, we used mfold to predict nucleotide changes that would not alter the overall secondary structure of the MicA molecule. The wild-type and the variant forms of MicA were cloned in a low copy number plasmid and were expressed from MicA own promoter. The synthetic MicA sRNAs were expressed *in trans* from a plasmid in cells deleted for the chromosomal *mic*A gene.The 5′ linear region of MicA is involved in the interaction with multiple target mRNAs [17]. MicA lacks a base pairing “seed” and the nucleotides involved in interactions with mRNAs can vary according to the target analysed. For this reason, we introduced extensive mutations in this 5′ linear sequence (MicA-5′mut) in order to maximize the disruption of MicA binding with its targets (Figure 1∶5′ end mutagenesis).

The proper RNA folding can also be crucial for interaction with mRNAs and MicA exhibits two stable stem-loops (Figure 1). Furthermore, such structured features can potentially act as stabilizing elements as they may serve as physical barriers against 3′-5′ exonucleolytic degradation [34]. These elements may thus play multifunctional roles and we constructed several mutants to study them. MicA-STEM1 mutant harbours mutations that almost disrupt completely the first stem-loop (located more closely to the 5′ end) without affecting the global conformation of the molecule. On the other hand, the second stem-loop (closer to the 3′ end) was shown to be thermodynamically stronger and we could not disrupt it as this greatly changed the secondary structure of MicA; hence mutations introduced in MicA-STEM2 were chosen to allow stem-loop relaxation while the overall secondary structure of the molecule was not disturbed. In addition to these single mutants we also constructed the double MicA-STEM1_2 mutant harbouring mutations in both stem-loops (Figure 1: Stem-loop mutagenesis).

Another module present in MicA RNA is the short linear A/U-rich region between the two stem-loops that is a predicted *in vitro* binding site for Hfq [14]. Base pairing between the sRNA and its target mRNA is facilitated in the presence of the RNA chaperone Hfq [26]. In order to analyse the *in vivo* importance of this region for the Hfq-dependent regulation of MicA we mutated the A/U-rich (5′-AAUUU-3′) to a C-rich stretch (5′-ACCAC-3′) as these mutations are predicted to almost disrupt Hfq-binding to the RNA (Figure 1: Hfq-binding site mutagenesis).

The last unit to be analysed here is the short poly(U) tail of the *rho*-independent terminator, a general feature of bacterial small RNAs. This was recently shown to be a key sequence for riboregulation and Hfq action on small RNAs [20], [28], [30]. Two mutants were designed for this region; MicA-3′mut1 exhibits two nucleotide substitutions in the poly(U) sequence while MicA-3′mut2 harbours more nucleotide changes (Figure 1∶3′ end mutagenesis). All the mutations described above were then analysed for their impact on MicA stability and capability to regulate its targets. All the work was performed with cultures in the stationary phase of growth, a condition in which MicA levels are increased [15], [38].

### 2. Experimental Determination of the Secondary Structure of MicA Mutants

**Figure 2:**
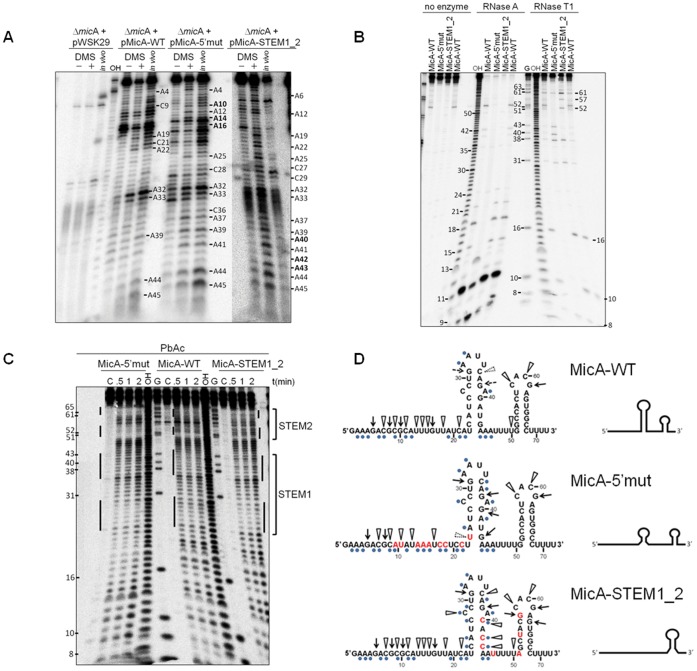
Determination of the secondary structure of MicA-5′mut and MicA-STEM1_2 RNAs. (A) Results from *in vitro* and *in vivo* dimethyl sulphate (DMS) modification assay. cDNA obtained from total RNA samples treated (+) or untreated (−) with DMS *in vitro*, as indicated on top of the gel. *In vivo* denotes the reactions from DMS added directly to cell cultures. The position of some adenosine and cytidine residues that reacted with DMS is given. RNA extracted from cells transformed with the empty pWSK29 vector or expressing the MicA-WT, MicA-5′mut RNA and MicA-STEM1_2 RNAs was tested. MicA-STEM1_2 is expressed to lower levels than MicA-WT (see Figure 7). Therefore, the panel with MicA-STEM1_2 RNA corresponds to the same gel but the image was slightly contrasted to better visualise the bands. The 3′ end of the MicA-DMS primer (Table III) used in the reverse transcription reaction is complementary to nucleotide 58 in MicA sequence. (B) Enzymatic probing of the different MicAs using RNase A and RNase T1. The position of several nucleotides is given. Controls with no addition of enzyme are shown on the left side of the gel. Alkaline ladders of MicA-WT are denoted as OH. A G-specific ladder generated by RNase T1 digestion of MicA-WT RNA under denaturing conditions is shown. Please note that in each series, MicA-WT was tested in duplicate. (C) Lead acetate probing. All reactions were done in native conditions, with addition of 5 mM PbAc. Incubation proceeded for 0.5, 1 or 2 minutes, as indicated. C is an untreated control, and G is a T1 ladder obtained under denaturing conditions. OH represents an alkaline ladder prepared with MicA-WT RNA. Some nucleotides are given for orientation. Thick lines on the side of the lanes represent the position of stem-loop arms. (D) Representation of MicA-WT, MicA-5′mut and MicA-STEM1_2 sequences, showing the enzymatic cleavages by RNase T1 (arrows) and RNase A (triangles) and the reactivity of nucleotides to DMS (blue dots below the nucleotides). Broken lines indicate weaker cleavage sites. Mutated nucleotides are shown in red. On the right side, it is given a schematic conformation of these RNAs.

Although mfold prediction was previously shown to agree with the structure of the wild-type MicA [15], [18], we decided to experimentally validate the structural models of two of our most relevant mutants, MicA-5′mut and MicA-STEM1_2 (Figure 2). To characterise *in vitro* and *in vivo* the RNA secondary structures of these two mutants, we have used a broad range of enzymatic and chemical probes. Namely, the techniques performed were dimethyl sulphate (DMS) modification of RNA nucleotides, the use of RNase A (cuts C and U unpaired residues) or RNase T1 (identifies unpaired G residues) and detection of single-stranded residues by lead acetate (PbAc) and in line probing.

First we carried out *in vitro* and *in vivo* probing with DMS, which methylates unpaired adenosines and cytidines. After DMS treatment, a specific antisense primer to the 3′ end of MicA was used to perform primer extension reactions. However, this amplification revealed to be highly problematic probably due to the strong STEM2 present in MicA secondary structure that prevented annealing of a primer in this region. In fact, we only succeeded in obtaining cDNA from cells expressing the MicA-STEM1_2 variant; this result seems to support the relaxation of the STEM2 secondary structure. Only the use of a modified LNA primer allowed amplification with good resolution from nucleotides 1 to 50. Complementary studies using additional methods allowed then a better resolution of the most 3′ end nucleotides.

The mfold prediction of the MicA-WT RNA structure was very accurate compared to our experimental data (Figure 2) and to what was previously reported to the chromosomally encoded wild-type MicA [15], [18]. A very good validation of the proposed computational model was also observed with the structure mapping of the MicA-STEM1_2. Mutations in the STEM1_2 MicA impaired the formation of STEM1 although mfold would predict the existence of a very weak stem-loop in this region; both methods agreed in the relaxation of the STEM2. Surprisingly, DMS probing and RNase cleavage assays of the MicA-5′mut RNA sugested that mutations introduced in the 5′ end resulted in the partial disruption of STEM1, a feature that was not predicted by the computational analysis. Using lead acetate (Figure 2C) and in line probing (Figure S2) we observed that disruption of STEM1 in the MicA-5′mut did not seem as stronger as observed in the MicA-STEM1_2 variant. Altogether, these results suggested that a relaxed STEM1 is still probably detected in the conformation of the MicA-5′mut RNA while no changes were detected on STEM2 conformation (Figure 2D).

### 3. The 5′ Domain Impacts MicA Stability through an RNase III-mediated Pathway

In order to study the impact of the different MicA modules on the stability of this sRNA, we measured the decay rate for the MicA-WT and compared it with the altered forms of MicA (represented in Figure 1).

We first focused on the analysis of the role of the 5′ end of MicA in the stability of this small RNA. Mutagenesis of the 5′ end of MicA was previously shown to have an effect in the regulation of its target mRNAs [15], [16], [39]. The 5′ mutant that we constructed harbours more extensive modifications (9 nucleotide changes) than the other reported mutants. The structure mapping of this RNA (Figure 2) revealed that modifications introduced in the 5′ end could also affect the conformation of the STEM1. However, the MicA-5′mut RNA is shown to act differently than the MicA-STEM1 variant that harbours mutations that disrupt the STEM1 but maintain the 5′ linear end intact (as presented in the following set of results). This indicates that mutations introduced in the 5′ end have an effect that can be separated from the presence of an intact STEM1.

**Figure 3:**
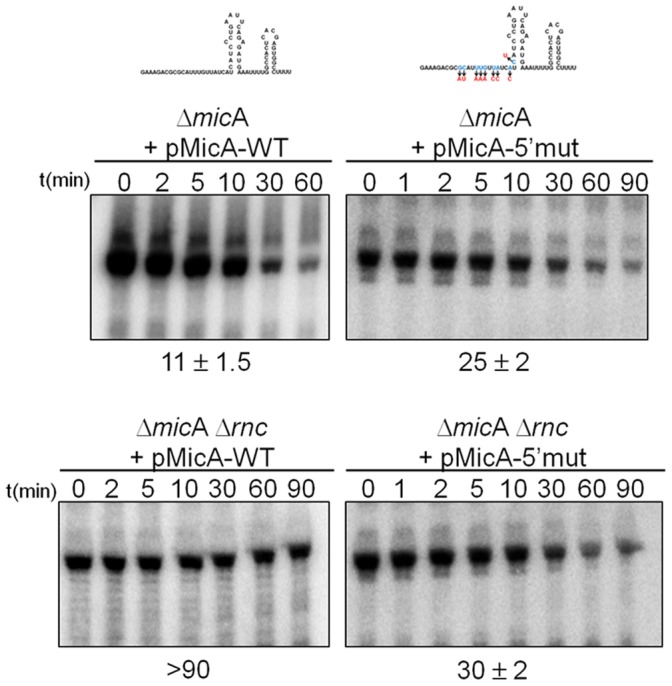
Mutagenesis of the 5′ linear domain of MicA. Northern blot analysis of MicA in Δ*mic*A cells or its isogenic derivative lacking RNase III (Δ*mic*A Δ*rnc*), expressing *in trans* either the wild-type MicA (from the pMicA-WT plasmid) or the 5′ mutated MicA variant (from the pMicA-5′mut plasmid). RNA was extracted from stationary phase cultures and MicA stability was measured as described in *Material and Methods*.

We found that MicA-5′mut is more stable than MicA-WT (half-lives of 25 min and 11 min, respectively as determined by Northern blotting) (Figure 3, upper panels). This result suggested that the degradation of MicA RNA is dependent on its 5′ end sequence. Since 5′ end of MicA interacts with target mRNAs, we could also infer from this result that the stability of MicA could also be linked to its ability to base pair with mRNAs.

RNase III is a double-stranded RNA endonuclease [40]. RNase III impact on MicA stability could result from the action on the small RNA itself (once it exhibits double-stranded regions) or from the activity of RNase III on the sRNA-mRNA hybrid. To test this, we performed an *in vitro* activity assay using purified *E. coli* RNase III and radioactive labelled wild-type MicA RNA as substrate and we found that this sRNA was not cleaved (Figure S3). This is in agreement with recent findings from our laboratory where it was shown that *Salmonella* RNase III (*rnc*) also does not cleave wild-type MicA *in vitro* but is able to cleave it when it is bound to its target *omp*A mRNA [41]. Consequently, the activity of RNase III against MicA can potentially be used as an indirect approach to indicate when MicA is base pairing with its target mRNAs.

To test the *in vivo* impact of *E. coli* RNase III in this regulation, we constructed the double mutant Δ*mic*A Δ*rnc* and measured the stability of the *trans*-encoded MicAs. As expected, MicA-WT was highly stabilized in the absence of RNase III (MicA barely decayed even after 90 min after blocking of transcription) (Figure 3, lower panels). The introduction of several mutations in the 5′ end of MicA strongly impaired the RNase III-mediated degradation of this sRNA; in the absence of RNase III, MicA-5′mut showed only a stability of 30 min *versus* the >90 min obtained for the MicA-WT. Hence, the 5′ end domain of MicA modulates this sRNA stability through an RNase III-dependent pathway.

Even though in the Δ*mic*A strain the MicA-5′mut is stabilised compared to the MicA-WT, we consistently observed that the MicA-5′mut is less abundant (Figure 3). The increasing stability that is observed is probably related to impaired RNase III activity against this molecule, as formation of MicA-mRNA target duplexes that corresponds to the RNase III substrate is suggested to be reduced. The lower abundance is more difficult to explain, but similar observations have been made [42], [43].

### 4. Distinct Roles of Stem-loops in Promoting MicA Stability

MicA displays two GC-rich stem-loops, STEM1 (immediately after the 5′ linear sequence) and STEM2 (closer to 3′ end) (Figure 1). Computational analysis using the mfold algorithm [18] predicts that STEM2 is thermodynamically stronger (ΔG = −12.0) than STEM1 (ΔG = −8.2). In agreement, our experimental structure mapping of the MicA variants showed that STEM1 conformation was strongly affected by the nucleotide changes introduced (Figure 2). Hence, we also wanted to analyse the importance of these structural elements in the control of MicA stability.

**Figure 4:**
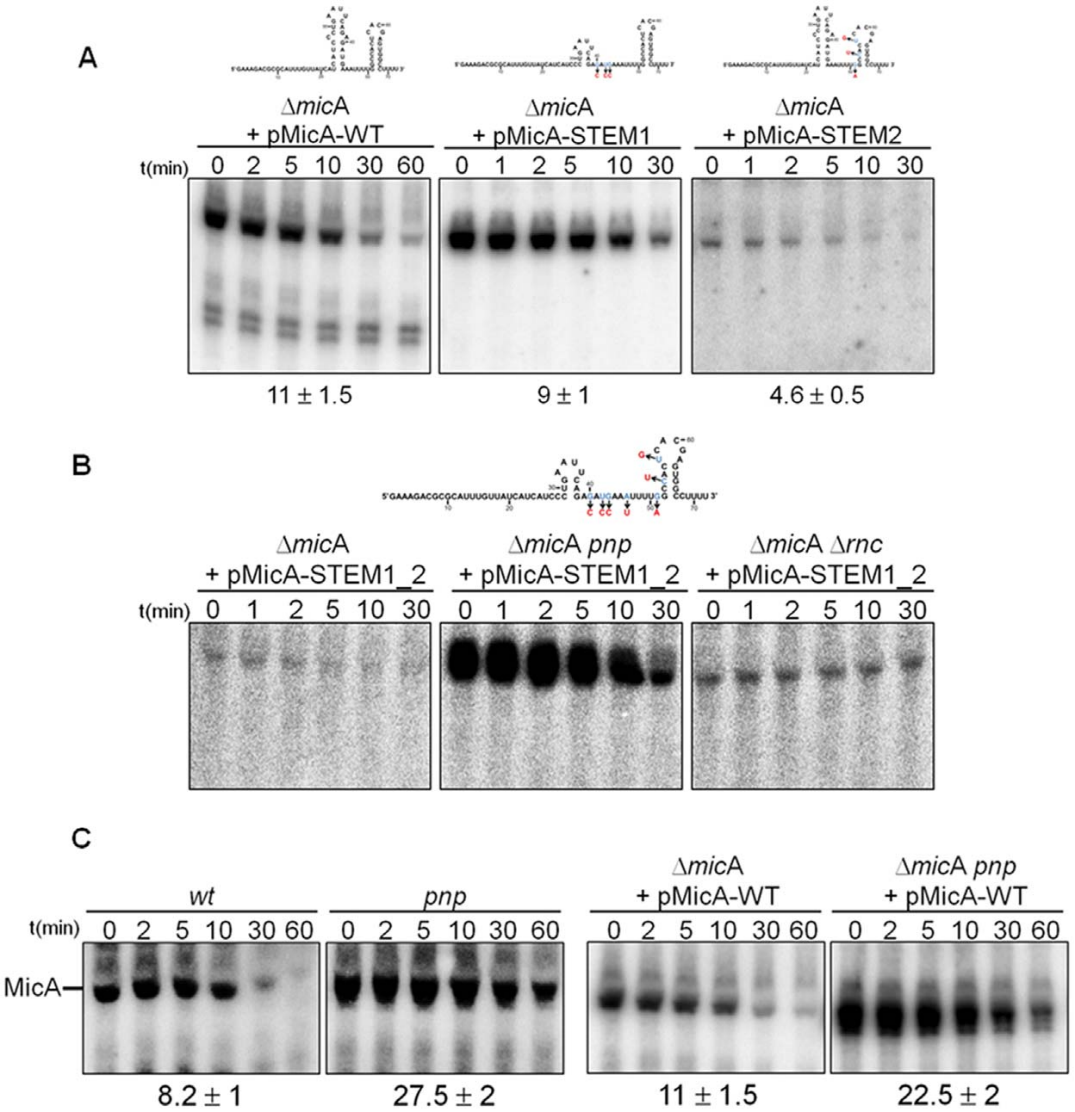
Mutagenesis of MicA stem-loops. (A) Northern blot analysis of MicA in Δ*mic*A cells expressing *in trans* either the wild-type MicA (from the pMicA-WT plasmid) or the stem-loops mutated MicA variants (from the pMicA-STEM1 or pMicA-STEM2 plasmids). When expressing the MicA-WT it is possible to visualise two lower molecular weight bands (<60 nts) that were previously identified in work performed in *Salmonella* to correspond to breakdown products of the duplex MicA-target mRNA [41]. (B) Impact of the disruption of both stem-loops (MicA-STEM1_2 variant) on MicA stability. Plasmid pMicA-STEM1_2 was used to transform Δ*mic*A cells and its isogenic derivatives lacking PNPase (Δ*mic*A *pnp*) or RNase III (Δ*mic*A Δ*rnc*). (C) Northern blot analysis of the chromosomally encoded MicA or the MicA-WT expressed from plasmid, comparing the stability pattern in cells expressing or not PNPase. RNA was extracted from stationary phase cultures.

Plasmids expressing either the wild-copy of MicA (pMicA-WT) or the altered STEM1 (pMicA-STEM1) or STEM2 (pMicA-STEM2) versions were used to transform Δ*mic*A cells. The expression and stability of these MicA variants were tested by Northern blotting (Figure 4A). Results showed that these structured elements play different roles in protecting MicA from degradation. Disruption of STEM1 barely affected MicA stability (MicA-STEM1 exhibit an half-life of 9 min) when compared to the MicA-WT (half-life of 11 min). Actually, the levels of the MicA-STEM1 variant are even slightly higher than the MicA-WT (Figure 4A). In contrast, perturbation of STEM2 considerably reduced the stability of this small RNA (MicA-STEM2 has a half-life of 4.6 min) as well as its abundance when compared to wild-type MicA (Figure 4A). In *Salmonella* two other point mutations located in STEM2 have been shown to affect the ability of MicA to downregulate *lam*B mRNA levels [16]. The MicA-STEM1_2 (harbouring mutations in both stem-loops) followed the results obtained with the single disruption of STEM2 (Figure 4B). Relaxation of the transcriptional terminator is likely to result in transcriptional read-through [32], [33]. However, as no major transcriptional termination read-through products were detected on these gels, the low levels of the MicA-STEM2 or the MicA-STEM1_2 variants are suggested to be consequence of their low stabilities. The 3′-5′ exonuclease PNPase was previously shown to degrade the wild-type MicA [12] and was the likely candidate for the rapid degradation of these MicA variants. A new Δ*mic*A *pnp* strain was then constructed and transformed with the adequate plasmid. Clearly, inactivation of PNPase resulted in a more long-lived MicA-STEM1_2 RNA and its levels were strongly increased (Figure 4B). The other major exoRNases (RNase II and RNase R) did not significantly participate in the degradation of this sRNA.

We also compared the activity of RNase III against the MicA-STEM1_2 RNA. This altered MicA variant with mutations on both hairpins was found to be stabilized in the Δ*mic*A Δ*rnc* when compared to the Δ*mic*A strain (Figure 4B). This was expected as MicA-STEM1_2 retains an intact 5′ end domain that is known to direct RNase III cleavage. Strikingly, the MicA-STEM1_2 expression level is much higher upon inactivation of PNPase rather than RNase III. This may suggest that a large fraction of MicA-STEM1_2 RNA might not be bound to its targets and therefore the free population of this sRNA is preferably degraded exonucleolytically by PNPase. As expected, PNPase was also confirmed to be important in the decay of the plasmid encoded MicA-WT (Figure 4C). Overall, these results indicate that STEM1 plays a minor role in protecting MicA while STEM2 functions as an effective stabilizer element protecting MicA from degradation.

### 5. The A/U-rich Linear Sequence is not the only Hfq-binding Site Present in MicA

Another structural domain in MicA is an A/U-rich single-stranded region flanked by the two stem-loop structures. Previous work showed that Hfq has *in vitro* affinity for this region [14]. However, the role of this module in regulation of MicA stability has not been addressed. In order to study this sequence, we constructed a plasmid expressing a MicA variant in which we changed the linear A/U-rich tail between the stem-loops to a C-rich sequence (Figure 1: Hfq-binding site mutagenesis). This is predicted to impair the binding of Hfq to this sequence [44]. The pMicA-hfq plasmid was then used to transform Δ*mic*A cells and RNA extracted from stationary phase cultures was analysed by Northern blotting and compared to Δ*mic*A harbouring pMicA-WT.

**Figure 5:**
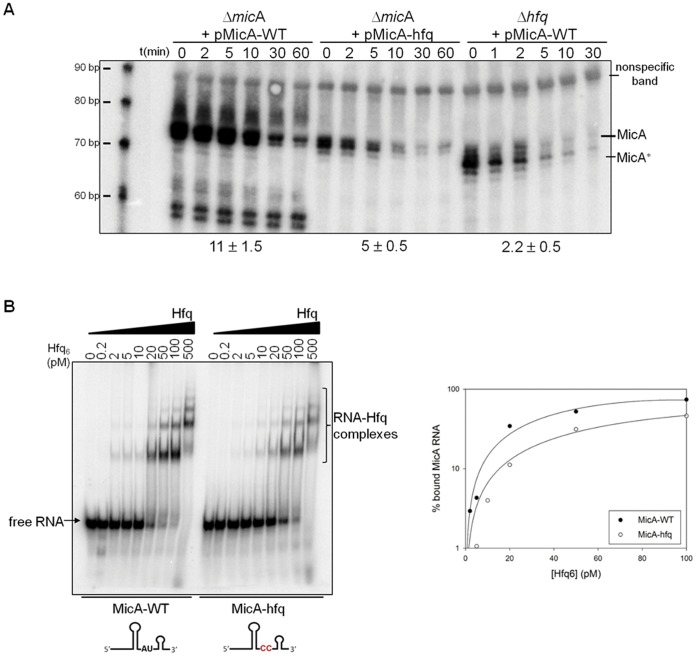
Mutagenesis of the A/U-rich domain, a Hfq-binding site in MicA. (A) Northern blot analysis of MicA in Δ*mic*A cells expressing *in trans* either the wild-type MicA (from the pMicA-WT plasmid) or a MicA variant with the A/U-rich domain mutated to a C-rich sequence (from the pMicA-hfq plasmid). Plasmid pMicA-WT was also used to transform a deletion strain of *hfq*. A smaller form of MicA (designated MicA∗) is only clearly observed in the absence of Hfq; this fragment had been previously identified [13]. A size marker is shown on the left of the gel. The riboprobe used to detect MicA, cross-reacts with a nonspecific band, (that is also detected on the Δ*mic*A strain, Figure S6) that was here used as loading control. A more stringent washing step eliminates this band without affecting the MicA signal, as previously described [12]. RNA was extracted from stationary phase cultures. (B) Mutagenesis of the A/U-rich domain of MicA to a C-rich sequence affects the Hfq binding ability to this small RNA. The gel mobility shift assay was performed with a constant amount of radiolabelled MicA-WT or MicA-hfq variant as RNA substrates and increasing amounts of purified Hfq protein, as indicated in the figure. The free RNA and the Hfq-RNA complexes are indicated. The gels were then dried and exposed to a PhosphorImager screen and quantified using ImageQuant software. The results were plot using SigmaPlot software and binding curves were fit. Filled circles represent MicA-WT and open circles represent MicA-hfq variant.

The mutated MicA-hfq is less stable (<50%) than the MicA-WT (Figure 5A). This clearly showed that the internal A/U-rich sequence plays relevant roles in determining MicA stability *in vivo*. If this sequence was the only Hfq-binding site present in MicA, the stabilities of both MicA-hfq in cells harbouring Hfq and the MicA-WT in cells lacking Hfq were expected to be similar. However, the MicA-WT in Δ*hfq* cells is less stable than the MicA-hfq variant in the presence of Hfq (Figure 5A).

Furthermore, the lack of Hfq results in the detection of a slightly smaller MicA species (MicA^∗^) (previously identified in [13]) that is not detected in the Δ*mic*A transformed with the pMicA-hfq plasmid (Figure 5A). Hfq is thus suggested to protect the 3′ end of MicA against nucleolytic degradation that originates the shorter and rather unstable MicA* species. Altogether, these results lead to the conclusion that the internal A/U-rich sequence is probably not the only Hfq-binding site in MicA. To confirm this, we have performed gel mobility shift experiments with a constant amount of radiolabelled MicA-WT or MicA-hfq variant as RNA substrates and increasing amounts of purified Hfq (Figure 5B). Hfq was able to form the same complexes with MicA-WT or MicA-hfq RNAs although binding with MicA-hfq was less efficient (with Kd values of 65 and 380 pM, respectively). Since Hfq could still form complexes with the MicA-hfq variant, this confirmed that this single-stranded A/U-rich sequence between the two stem-loops is not the only Hfq-binding site present in MicA.

### 6. The 3′ U-rich Terminator Sequence is Critical for MicA Stability

Hfq binds preferably A/U-rich sequences but was also shown to interact with other U-rich elements present in mRNAs [45]. The poly(U) tail from the transcriptional terminator was thus an excellent candidate to interact with Hfq (Figure 1). Recent findings demonstrated that the poly(U) sequence downstream the terminator is involved in the Hfq-dependent regulation of small RNAs [20], [28]. We further analysed the role of this domain in the control of MicA stability.

**Figure 6:**
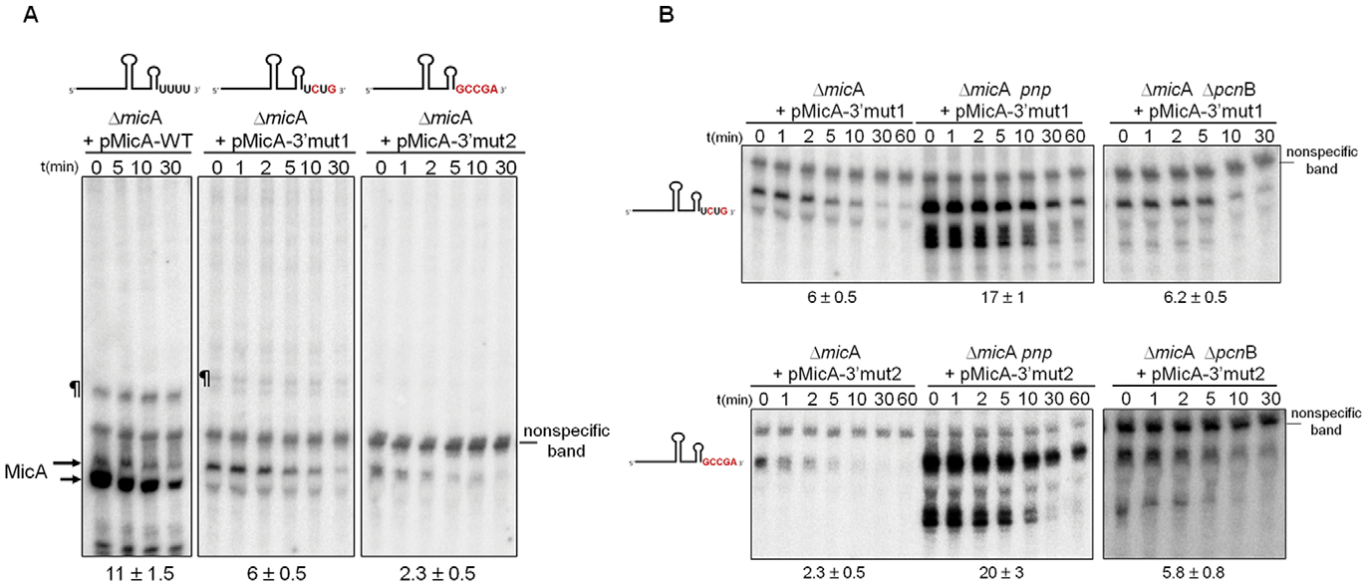
Mutagenesis of the 3′ end U-rich domain of MicA. (A) Effect of mutations in the 3′end U-rich linear sequence in the stability of the MicA RNA. Northern blot analysis of MicA in Δ*mic*A cells expressing *in trans* the wild-type MicA (from the pMicA-WT) or the mutated 3′ end variants (from the pMicA-3′mut1 or pMicA-3′mut2 plasmids). Read-through bands are indicated by the symbol (¶). Two different sized forms of MicA can be detected and are marked with arrows on the side of the gel. (B) Northern blot analysis of MicA in Δ*mic*A cells or its derivative isogenic mutants lacking either PNPase (Δ*mic*A *pnp*) or Poly(A) polymerase I (Δ*mic*A Δ*pcn*B) expressing *in trans* either the mutated MicA-3′mut1 or the MicA-3′mut2 variant. RNA was extracted from stationary phase cultures. Upon hybridization of the membrane, a nonspecific band is observed and was here used as loading control [12].

We constructed two MicA mutants in which we changed the nucleotides in the 3′ poly(U) tail. In the MicA-3′mut1 variant, the 5′-UUUU-3′ linear sequence immediately after the terminator was changed to a 5′-UCUG-3′ sequence while in the MicA-3′mut2 this modification was more extensive (to 5′-GCCGA-3′) (Figure 1∶3′ end mutagenesis). The corresponding plasmids harbouring these nucleotide changes in the 3′ end of MicA were used to transform the Δ*mic*A strain. In order to attenuate inefficient transcription termination that could arise from modification of the 3′ poly(U) sequence, an additional stretch of 8 T’s was included immediately after the 4U residues in both cloning strategies. The MicA-3′mut1 variant showed decreased stability when compared to the wild-type (half-life from 11 min to 6 min) (Figure 6A). Extended mutagenesis of the 3′ RNA sequence even resulted in a more drastic reduction (to 2.3 min for MicA-3′mut2).

The 3′ altered MicA’s are slightly longer than the wild-type MicA (Figure 6A). However, the elongated RNA species was also detected (in low level) when expressing the MicA-WT (Figure 6A, upper arrow on side of the gel). The wild-type MicA terminates in a linear stretch of 4 U’s which functions as efficient termination site [15], [17], [38]. Nevertheless, an alternative transcriptional termination site located next to the this sequence can be found in the *mic*A DNA (5′-TTTTCTTTT-3′) which can lead to slightly longer MicA species, as we observe in Figure 6A. Mutagenesis of the poly(U) sequence most probably results in the relaxation of the transcriptional termination leading to increasing amounts of this read-through RNA. However, even after the extensive modification of the 3′ poly(U) sequence in the MicA-3′mut2 we did not detect in these gels the accumulation of other major transcriptional read-through bands when compared to the MicA-WT (Figure 6A). This suggests that the reduced levels of the 3′ altered MicA RNAs are mainly consequence of its rapid turnover, as observed with the mutants in STEM2 (Figure 4). In fact, we found that inactivation of PNPase resulted in the strong accumulation and stabilization of both the 3′ MicA modified RNAs (Figure 6B).

In contrast, inactivation of the poly(A) polymerase (encoded by the *pcn*B gene) [46] did not show a great effect in the degradation of these full-length 3′ end MicA mutants (Figure 6B) although MicA-3′mut2 RNA was more affected than the MicA-3′mut1 variant. Yet, polyadenylation seemed to affect the degradation of an intermediate breakdown product (Figure 6B). Inactivation of PNPase resulted in much stronger stabilisation of these sRNAs, an indication that PNPase is not dependent on an active poly(A)-dependent pathway to actively degrade these small RNAs.

### 7. Differential Control of Target mRNAs by MicA Variants

We wanted to investigate whether our different MicA mutants were functional in riboregulation. To test this, we analysed the RNA levels of three different targets: *omp*A, *tsx* and *ecn*B mRNAs. OmpA is a major outer membrane protein and was the first identified target of MicA. EcnB (entericidin B membrane lipoprotein) and Tsx (nucleotide transporter) were recently shown to be specifically regulated by MicA in *Salmonella* [14]–[17]. Our work extends the list of known target mRNAs in *E. coli* as for the first time *tsx* and *ecn*B transcripts are also shown to be regulated by MicA.

**Figure 7:**
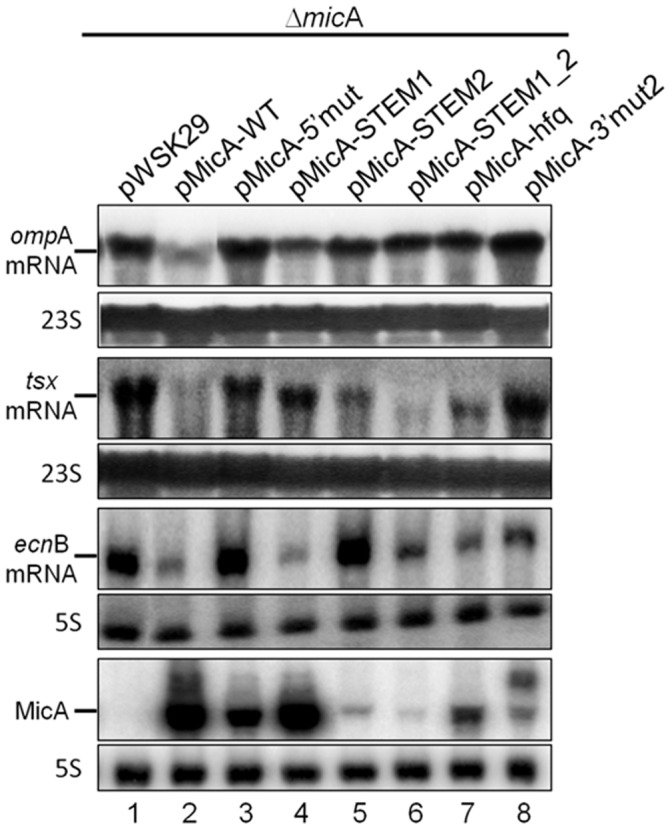
Differential control of target mRNAs by the synthetic MicA variants. Northern blot analysis of *omp*A, *tsx*, *ecn*B and MicA transcripts. Total RNA was extracted from stationary phase cultures of Δ*mic*A cells transformed with a low copy number based plasmid (pWSK29) expressing either the wild-type copy of MicA or one of the mutated variants described in this work. Plasmids used were: pWSK29 (lane 1), pMicA-WT (lane 2), pMicA-5′mut (lane 3), pMicA-STEM1 (lane 4), pMicA-STEM2 (lane 5), pMicA-STEM1_2 (lane 6), pMicA-hfq (lane 7) and pMicA-3′mut2 (lane 8). 23S was used as loading control for the *omp*A and *tsx* transcripts (analysis from agarose Northern blots) while 5S was used as loading control for *ecn*B and MicA RNAs (analysis from polyacrylamide Northern blots).

A deletion strain of *mic*A was transformed either with the empty plasmid (pWSK29), the plasmid expressing the wild-type copy of MicA or with the plasmids harbouring the different MicA variants and the levels of the target mRNAs were then evaluated. This provided a simple approach to test how the different mutations were affecting MicA repressor activity. Overexpression of the wild-type MicA is very efficient in the downregulation of its target mRNAs as compared to Δ*mic*A transformed with the empty vector (compare Figure 7, lane 1 and lane 2). On the other hand, MicA mutants exhibited different levels of repression (Figure 7A, lanes 3–8).

Compared to the MicA-WT expression, the mutated forms of MicA (with exception of MicA-STEM1) present in Figure 7 have reduced concentrations as result of their lower stabilities (Figure 7, lower panel) and this could contribute to different levels of repression observed. Nevertheless, despite this different expression levels, we verified that a same MicA variant can differentially affect the levels of different target mRNAs. Even though the levels of MicA-5′mut were lower than the MicA-WT, all the target mRNAs accumulated showing that modification of the 5′ linear sequence is critical to downregulate the expression of these targets (compare Figure 7, lane 2 and lane 3). In addition, the levels of MicA-STEM1 were identically to MicA-WT but we could detect different regulatory effects as MicA-STEM1 RNA was still able to repress *ecn*B expression (identically to MicA-WT) while it did not function as well to downregulate *omp*A or *tsx* mRNA levels (compare Figure 7, lanes 2 and 4). Conversely, the levels of MicA-STEM2 were strongly reduced but MicA-STEM2 was found to be much more important in the regulation of *ecn*B and *omp*A mRNAs than STEM1 (compare Figure 7, lane 2 with lanes 4–5).

The stem-loops are shown to have different effects on the regulation of different targets. STEM2 seems to have a more generalised effect, affecting all the tested mRNAs while STEM1 was shown to particularly affect the *tsx* mRNA (compare Figure 7, lane 2 with lanes 4–5). MicA-STEM1_2 variant (harbours mutations in both MicA stem-loops) was not found to simply add the effects of STEM1 and STEM2 mutations (compare Figure 7, lanes 4–6). Despite the low levels of MicA-STEM1_2 (that follows the expression levels found to MicA-STEM2), this sRNA is still able to repress the expression of *omp*A and *ecn*B mRNAs but unlike MicA-STEM1 it did not seems to repress *tsx* expression (compare Figure 7, lanes 4–6).

The mutants for the two high affinity Hfq-binding sites in MicA were also analysed for their riboregulatory activity (Figure 1 and Figure 7, lanes 7–8). Mutagenesis of either the internal A/U-rich or the 3′ poly(U) tail reduces the ability of Hfq to interact with the sRNA and this is expected to affect the base pairing ability of the sRNA with the target mRNAs [28], [30]. MicA-hfq variant was less functional than the MicA-WT in the downregulation of all the targets, showing that this domain is required for riboregulation. However, this was shown to be more important for regulation of *omp*A mRNA than for the others transcripts tested (Figure 7, compare lanes 2 and 7). On the other hand, the MicA-3′mut2 variant (harbouring mutations in the 3′ poly(U) tail) resulted in the accumulation of all the targets analysed (Figure 7, compare lanes 2 and 8), to a level higher than the one obtained when overexpressing the MicA-hfq variant (Figure 7, compare lanes 7 and 8). These results suggest that the 3′ poly(U) sequence plays more relevant roles than the internal A/U-rich sequence in promoting interactions with the different target mRNAs; it is also possible that 3' poly(U) sequence can affect specificity of a sRNA.

## Discussion

The architecture of a small RNA is critical for its stability, influences the formation of sRNA-protein complexes and can affect base pairing with target mRNAs. The knowledge of the important factors controlling the sRNA abundance in the cell is of utmost importance for the manipulation of sRNA-based pathways. Through our mutagenic studies we have defined distinct modules in MicA that were shown to play distinct roles in protecting MicA from degradation. Mutations in the 5′ end domain resulted in the stabilisation of this sRNA, presumably by impairing RNase III activity against MicA. On the other hand, mutations in several 3′ end elements resulted in unstable MicA’s and we showed that the 3′-5′ exonuclease PNPase was a major player in this degradation. Our data also suggest that different domains of MicA can be involved in the riboregulation of target mRNAs. Moreover, we have shown that the effect of these sRNA mutations in their regulatory pathways cannot be directly deduced from the levels or stability of the small RNAs.

The free form of MicA is not cleaved by RNase III and only when it is bound to a target mRNA it becomes a substrate to RNase III (Figure S3). This also helps explaining why mutagenesis of the 5′ end domain of MicA resulted in the stabilisation of this sRNA (Figure 3). This implies that the free (not bound to its targets mRNAs) population of MicA is degraded by a distinct pathway that does not necessarily involve RNase III. In fact, our data suggest that PNPase is actively involved in this decay. Therefore, alternative degradation pathways are used to control either the free MicA or the target-bound MicA. In an apparent paradox, even though MicA-5′mut was found to be more stable it is less abundant than MicA-WT (Figure 3). Similar results can be found in the literature for other RNAs. In fact, in *E. coli* the levels of RNAs not always have a direct correspondence to their half-lives as shown by a microarray analysis [42]. We may speculate that mutagenesis of the 5′ linear sequence of MicA could affect the production of this sRNA. Interestingly, it has been suggested that mutations inserted in the 5′ UTR sequence can influence the transcription rate [47].

The linear 5′ end sequence was shown to be the main domain that many small RNAs use to bind their targets (for example RybB, OmrA, OmrB and MicA) [7], [17], [19], [21]. We confirmed that the 5′ linear sequence of MicA (corresponding to the first 23 nts) is essential for repression of all the targets analysed (*omp*A, *tsx* and *ecn*B mRNAs). From these, *tsx* mRNA was the less affected by the mutations in the 5′ end of MicA, probably because the nucleotide changes still resulted in an extensive complementation with this target, as predicted by computational analysis (Figure S4). However, we have found that the 5′ linear sequence of MicA does not seem to be the only domain involved in the regulation of the targets (Figure 7).

We observed that MicA stem-loops could affect differently the amounts of the distinct target mRNAs. STEM2 was found to be much more important for the *in vivo* repression of both *omp*A and *ecn*B mRNAs than STEM1, unlike we could expect from the RNAhybrid prediction (Figure S4). In contrast, STEM1 was critical for regulation of *tsx* transcript levels while disruption of STEM2 had a considerably less impact on this mRNA. MicA-STEM1_2 is clearly less efficient in the repression of *tsx or ecnB* mRNA levels (Figure 7). Therefore, these results suggest that the structure of both MicA stem-loops is important for interaction with such targets.

The main function of stem-loops is usually considered the protection of RNA against degradation. The formation of a double-stranded region within the sRNA can sequester sequences susceptible to RNase E endonucleolytic cleavages and efficiently act as physical barriers against 3′-5′ exonucleolytic degradation [40]. The two stem-loops present in MicA were found to play distinct roles. Surprisingly, the extensive disruption of STEM1 did not significantly affect the stability of MicA (Figure 4A). The main role of STEM1 seems thus not to be the protection of the sRNA against degradation, unlike it was shown to happen with STEM2. This has implications in the relative abundance of these variants as MicA-STEM1 shows identical levels to MicA-WT whereas MicA-STEM2 is strongly downregulated (Figure 7). However, as showed here, some targets like *omp*A and *ecn*B mRNAs are more strongly affected by MicA-STEM2 than by the MicA-STEM1 variant, which supports that the effects of these mutations in the regulatory pathways are not simply the result from changes in sRNA stability. The Hfq-binding sites in MicA are in two separate domains: the internal A/U-rich sequence and the 3′ U-rich tail after the transcriptional terminator. The Hfq-binding site mutants of MicA were expected to be less efficient in the interactions with target mRNAs (because Hfq is known to accelerate the rate of sRNA-mRNA duplex formation) [26]. Surprisingly, our results showed that these sequences can play distinct roles in the regulation of the different targets. Mutation of the 3′ U-rich sequence of MicA was shown to have a more generalised effect in all the mRNAs tested while mutation of the A/U-rich sequence had a more pronounced impact in the regulation of *omp*A mRNA levels than on the other targets. RNAhybrid predicted that mutations affecting the 3′ poly(U) sequence of MicA would affect more the base pairing with target mRNAs but failed to predict the impact of disrupt the internal A/U-rich sequence of MicA in the regulation of *omp*A mRNA levels (Figure S4).

These results suggest that interaction of Hfq with the sRNA seems to greatly depend on the target itself or might require the interaction with additional factors. Mutants in one of the high affinity Hfq-binding sites of MicA (MicA-hfq and MicA-3′mut variants) were shown to accumulate at levels inferior to the MicA-WT (Figure 7). However, for each mutation we can observe distinct effects for the different targets. For example, changes introduced in the 3′ U-rich linear sequence of MicA affected more strongly the levels of *omp*A and *tsx* mRNA in comparison to *ecn*B mRNAs. Again we conclude that this differential response shows that the effect of the mutations on target expression cannot be simply deducted from the sRNA levels.

Hfq is known to protect sRNA from RNase E and PNPase-mediated degradation [13], [31]. Mutations that disrupted the Hfq-binding sites were found to result in more labile MicA’s probably because these MicA variants become more accessible to the action of RNases. Modification of the 3′ U-rich linear tail of the transcriptional terminator (Figure 6) was shown to destabilize MicA to a higher extension than the mutations introduced in the internal A/U-rich sequence (Figure 5). Stability measurements revealed that PNPase was the main enzyme involved in the degradation of these MicA variants. We also examined the effect of modifying the 3′ poly(U) tail of the RybB, a small RNA that shares multiple targets with MicA [17], [19], [21]. Modification of the nucleotides immediately after the terminator stem-loop (from poly(U) to a 5′-CCGUC-3′ sequence) resulted as well in a more labile sRNA (Figure S5). Recent findings showed that shortening of the 3′ U-rich tail of other sRNAs also resulted in the instability of these sRNAs [20]. Therefore, Hfq binding to the uridylated 3′ end of MicA agrees with the protection of this protein against the 3′-5′ exonucleolytic degradation by PNPase (this work and [13]).

The modular structure of MicA helps explaining the dynamics of interaction with its multiple targets. The 5′ end of MicA is critical for repression of some targets (such as *omp*A expression) while the 3′ end of MicA harbours elements that may be more relevant for regulation of other targets (such as *tsx* mRNA). These findings can most probably be extended to other regulatory RNAs, judging by the results of a computational search which predicted that several other small RNAs can potentially use different regions to establish base pairing interactions with their targets [48]. FnrS and Spot42 are good examples of this, as both were shown to use different single-stranded regions for base pairing with different set of targets [49], [50].

Our work confirms the importance of the 5′ end domain both in riboregulation and in the stabilisation of the sRNA. However, we expand this view by showing that 3′ end elements not only are critical for the stability of the sRNA but are also suggested to be involved in the regulation of some target mRNAs. As a matter of fact, the 3′ end is shown to harbour different stabilizer elements, namely stem-loops and high affinity Hfq-binding sites. Actually, Hfq has even a higher affinity for the 3′U-rich sequence rather than for internal A/U-rich sequences typically found in small RNAs [28], [30]. The 3′ end terminal nucleotides of MicA are highly conserved (as observed in the sequence alignment in Figure 1) and most likely our findings with *E. coli* MicA can be extrapolated across species. Moreover, the modular structure of MicA is commonly found among small RNAs, supporting that our results may be generalized to other non-coding RNAs [19]–[22]. There has been growing interest in the use of synthetic regulatory RNAs to program gene expression networks [51]–[53]. We believe that mutations that alter the 3′end region, namely the 3′U-rich sequence of the sRNA can be a useful strategy to manipulate the networks regulated by small RNAs.

## Materials and Methods

### Strains and Growth Conditions

*E. coli* K-12 strain MG1693 [54] or its derivatives were used in this work (Table 1). Deletion mutant of *ryb*B was constructed by the one step inactivation of chromosomal genes method [55]. Bacteria were grown at 37°C in Luria-Bertani (LB) medium supplemented with thymine (50 µg ml^−1^). SOC medium was used to recover cells after heat-shock in plasmid transformation steps. When required, antibiotics were present at the following concentrations: chloramphenicol, 50 µg ml^−1^, kanamycin, 50 µg ml^−1^; ampicillin, 100 µg ml^−1^.

**Table 1:** Strains used in this work.

Strain	Relevant genotype	Reference
**GSO80**	MC4100*hfq*	[67]
**MG1693**	*thy*A715	[54]
**SK5691**	*thyA715 pnp*7	[54]
**SK7622**	*thyA715* Δ*rnc*38	[68]
**CMA413**	*thyA715* Δ*mic*A	[12]
**CMA428**	MG1693 *hfq*	[13]
**CMA514**	*thyA715* Δ*mic*A+pMicA-WT	This study
**CMA515**	*thyA715* Δ*mic*A+pMicA-5′mut	This study
**CMA516**	*thyA715* Δ*mic*A+pMicA-STEM1	This study
**CMA517**	*thyA715* Δ*mic*A+pMicA-STEM2	This study
**CMA518**	*thyA715* Δ*mic*A+pMicA-STEM1_2	This study
**CMA519**	*thyA715* Δ*mic*A+pMicA-hfq	This study
**CMA520**	*thyA715* Δ*mic*A+pMicA-3′mut1	This study
**CMA521**	*thyA715* Δ*mic*A+pMicA-3′mut2	This study
**CMA522**	*thyA715* Δ*mic*A Δ*rnc*38+ pMicA-WT	This study
**CMA523**	*thyA715* Δ*mic*A Δ*rnc*38+ pMicA-5′mut	This study
**CMA524**	*thyA715* Δ*mic*A *pnp*7+ pMicA-WT	This study
**CMA525**	*thyA715* Δ*mic*A *pnp*7+ pMicA-STEM1_2	This study
**CMA526**	*thyA715* Δ*mic*A Δ*rnc*38+ pMicA-STEM1_2	This study
**CMA527**	*thyA715* Δ*mic*A Δ*pcn*B+pMicA-3′mut1	This study
**CMA528**	*thyA715* Δ*mic*A Δ*pcn*B+pMicA-3′mut2	This study
**CMA529**	MG1693 *hfq*+pMicA-WT	This study
**CMA530**	*thyA715* Δ*mic*A *pnp*7+ pMicA-3′mut1	This study
**CMA531**	*thyA715* Δ*mic*A *pnp*7+ pMicA-3′mut2	This study
**CMA532**	*thyA715* Δ*ryb*B+pRybB-WT	This study
**CMA533**	*thyA715* Δ*ryb*B+pRybB-3′mut	This study

### Construction of Plasmids

All plasmids used in this work are based on the very low copy number pWSK29 [56] and are indicated in Table 2. DNA fragments containing the mutagenic *mic*A variants (MicA-5′mut, MicA-STEM1, MicA-STEM2, MicA-STEM1_2, MicA-hfq, MicA-3′mut1, MicA-3′mut2) were amplified by PCR overlapping using the oligonucleotides indicated in Table III. Partial fragments were amplified with MicA-*Hind*III and the respective mutagenic forward primer or MicA-*Pst*I and the respective mutagenic reverse primer (Table III). PCR bands were gel eluted using the gel extraction NucleoSpin Extract II kit (Macherey-Nagel). For each mutation, the partial PCRs carry an overlapping region of 20 nucleotides. Approximately equal amounts of each partial PCR (for a given construct) were added to *Pfu* reaction mix containing dNTPs but lacking primers. The extension step (30 s at 95°C, 60 s at 55°C and 30 s at 72°C) proceeded for 15 cycles. The external primers MicA-*Hind*III and MicA-*Pst*I were then added and the PCR reactions run for 20 cycles. A DNA fragment (274 bp) encompassing the entire wild-type MicA (MicA-WT) was directly amplified using primers MicA-*Hind*III and MicA-*Pst*I. All DNA inserts include the MicA natural promoter (previously identified in [57] and were *Hind*III/*Pst*I cloned into pWSK29. Competent DH5α cells were used in the cloning procedure. Positive clones were selected by colony PCR. The nucleotide sequences of all constructs were confirmed by DNA sequencing (Stab Vida).

**Table 2:** Plasmids used in this work.

Plasmid	Comments	Reference
**pWSK29**	very low copy number plasmid; ampicillin resistance	[56]
**pMicA-WT**	wild-type copy of MicA	This study
**pMicA-5′mut**	MicA variant harbouring mutations in the 5′ linear sequence	This study
**pMicA-STEM1**	MicA variant harbouring mutations in stem-loop 1	This study
**pMicA-STEM2**	MicA variant harbouring mutations in stem-loop 2	This study
**pMicA-STEM1_2**	MicA variant harbouring mutations in stem-loops 1 and 2	This study
**pMicA-hfq**	MicA variant harbouring mutations in the internal A/U-rich linear sequence (an high affinity Hfq-binding site)	This study
**pMicA-3′mut1**	MicA variant harbouring 2 nucleotides changes in the 3′ end U-rich terminator sequence	This study
**pMicA-3′mut2**	MicA variant harbouring 5 nucleotides changes in the 3′ end U-rich terminator sequence	This study
**pRybB-WT**	wild-type copy of RybB	This study
**pRybB-3′mut**	MicA variant harbouring 5 nucleotides changes in the 3′ end U-rich terminator sequence	This study

### RNA Extraction and Northern Analysis

For decay experiments, blocking of transcription was obtained by adding rifampicin to a final concentration of 500 mg ml^−1^. Culture samples were withdrawn at defined timepoints thereafter and mixed with an equal volume of RNA stop buffer (10 mM Tris at pH 7.2, 5 mM MgCl_2_, 25 mM NaN_3_, and 500 mg ml^−1^ chloramphenicol). Total RNA was extracted by the phenol:chloroform method from stationary phase cultures as previously described [13]. Genomic DNA was removed from samples using the Turbo DNase (Ambion). For Northern analysis, 10–30 µg of total RNA was fractionated under denaturing conditions either in 6% (for detection of the *ecn*B mRNA) or 10% polyacrylamide/7 M urea gels in TBE (for detection of the sRNAs) or by 1.2% agarose formaldehyde-denaturing gel in MOPS buffer (for detection of *omp*A and *tsx* mRNAs). RNAs were transferred onto Hybond-N^+^ membrane (GE Healthcare) and U.V. crosslinked by UV irradiation using a UVC 500 apparatus (Amersham Biosciences). Membranes were hybridized with radiolabelled specific probes overnight in PerfectHyb Plus Hybridization Buffer (Sigma Aldrich) at 42–68°C. Specific probes were obtained either by 5′ end-labelling of antisense oligonucleotides using ^hello^γ-^32^P]-ATP and T4 polynucleotide Kinase (Fermentas) or by *in vitro* transcription reactions with PCR DNA templates carrying a T7 promoter sequence through the use of ^hello^α-^32^P]-UTP and T7 RNA polymerase (Promega). Radiolabelled probes were purified on G25 Microspin columns (GE Healthcare). The probe used to detect MicA in the Northern blot experiments correspond to an antisense riboprobe complementary to the entire wild-type MicA sequence (about 74 nucleotides in length). RNA was analysed by Phosphorimaging (Storm860) using the ImageQuant software (Molecular Dynamics). The half-lives of RNA were determined by linear regression using the logarithmic of the percentage of RNA remaining *versus* time, considering the amount of RNA at 0 min as 100%. Primers used in this work were obtained from Stab Vida (Portugal) and are described in Table 3. All radiochemicals were purchased from PerkinElmer.

**Table 3:** Primers used in this work.

PRIMER	Sequence (5′-3′)
**Overlapping PCR**	
**MicA-HindIII**	AATGGAAGCttCTGATACCGAACCG
**MicA-PstI**	TTTTCGCCACCCGAACTGCAGGC
**MicA-5'mut F**	GCATATAAATCCTCCTTATCCCTGAATTCAGAGATGAAATTTTGGC
**MicA-5'mut R**	GATAAGGAGGATTTATATGCGTCTTTCATATACTCAGACTCGCCT
**MicA-STEM1 F**	GAcAccAAaTTTTgGCcACtCACGAGTGGCCTTTTTCTTTTCTGTCAGG
**MicA-STEM1 R**	aGTgGCcAAAAtTTggTgTCTGAATTCAGGGATGATGATAACAAATGCGC
**MicA-STEM2 F**	GAgAtgAAaTTTTaGCtACgCACGAGTGGCCTTTTTCTTTTCTGTCAGG
**MicA-STEM2 R**	cGTaGCtAAAAtTTcaTcTCTGAATTCAGGGATGATGATAACAAATGCGC
**MicA-STEM1_2 R**	cGTaGCtAAAAaTTggTgTCTGAATTCAGGGATGATGATAACAAATGCGC
**MicA-STEM1_2 F**	GAcAccAAtTTTTaGCtACgCACGAGTGGCCTTTTTCTTTTCTGTCAGG
**MicA-hfq F**	CAGAGATGAAccacTGGCCACTCACG
**MicA-hfq R**	GTGGCCAGTGGTTCATCTCTGAATTCAGGGATG
**MicA-3'mut1 F**	aaaaAAAAGtcggcAGGCCACTCGTGAG TGGCC
**MicA-3'mut1 R**	TgccgaCTTTTttttCTGTCAGGCGTGTTTTTCCAG
**MicA-3'mut2 F**	aaaaAAAAGcAgAAGGCCACTCGTGAGTGGCC
**MicA-3'mut2 R**	TTCTGCTTTTttttCTGTCAGGCGTGTTTTTCCAG
**RybB-PstI**	CGTCCTGCaGACGCTGGCAGGGACAATC
**RybB-HindIII**	GACCGTAAGCttCTATCGCGCGAGGAG
**RybB-3mut F**	GTTGATGGGTgccTcTTTTTTTTGTTATCTAAAACTTATC
**RybB-3mut R**	GATAACAAAAAAAAgAggcACCCATCAACCTTGAACCG
**RNA substrate (** ***in vitro*** ** transcription)**	
**T7-MicA**	TAATACGACTCACTATA GAAAGACGCGCATTTGTTATCATC
***in vitro*** ** MicA-wt**	AAAAGGCCACTCGTGAGTGGC
***in vitro*** ** MicA-mut2**	TCGGCAGGCCACTCGTGAGTGGCC
**cDNA synthesis (reverse transcriptase reaction)**	
**MicA-DMS LNA**	AAA+A+G+AA+A+A+AGGCCACTCGTG
**Gene deletion**	
**RybB-delFw**	CACAACCGCAGAACTTTTCCGCAGGGCATCAGTCTTAATTAGTATTGTGTAGGCTGGAGCTGCTTC
**RybB-delR**	TGGTTGAGAGGGTTGCAGGGTAGTAGATAAGTTTTAGATAACGGTCCATATGAATATCCTCCTTAG
**Northern probes**	
**MicA-T7**	TAATACGACTCACTATAG GAAGGCCACTCGTGAGTGGCCAA
**MicA-Fw**	GAAAGACGCGCATTTGTTATC
**RybB-T7**	TAATACGACTCACTATAG GAACAAAAAACCCATCAACCTTGAACCG
**RybB-Fw**	ACTGCTTTTCTTTGATGTCCC
***omp*** **A-T7**	TAATACGACTCACTATAGG AAAAAAAACCCCGCAGCAGC
***omp*** **A-Fw**	TTGTAGACTTTACATCGCCAGGG
***ecn*** **B-T7**	TAATACGACTCACTATAGG TTATTGCTGCGCTTTCGTTGC
***ecn*** **B-Fw**	ATGGTGAAGAAGACAATTGCAGCG
***tsx-*** **T7**	TAATACGACTCACTATAG GGCTCATCGGCAGGCCAGTGTCG
***tsx-*** **Fw**	GCGGTACTGGCGCTCTCTTCG
**23S-RNA**	CCT ACA CGC TTA AAC CGG GAC
**5S-RNA**	CAT CGG CGC TAC GGC GTT TCA CTT C

Nucleotide changes are indicated in small capitals;

T7 promotor sequence is underlined.

“+” precedes LNA-modified nucleotides.

### Electrophoretic Mobility Shift Assays (EMSA)

Binding assays were performed in 10 mM Tris–HCl (pH 8), 1 mM EDTA, 80 mM NaCl and 1% glycerol (v/v) [58] with increasing concentrations of His_6_-tagged Hfq purified protein (kindly provided by Eliane Hajnsdorf) and a constant amount of radiolabeled [α-^32^P]-UTP MicA as substrate. Reactions were incubated at 37°C for 30 min. EMSA samples were then electrophoresed on native 5% polyacrylamide gels in 1× TBE buffer in a cold room. Dried gels were then exposed on Phosphorimager screens and the corresponding signals were analysed using the ImageQuant software (Molecular Dynamics). Binding data were fit to a Polynomial Quadratic curve and Kd values were calculated from the fit of the curve using SigmaPlot software (Systat Software).

### 5′-end Labelling of RNA

MicA-WT and MicA-mutant RNA variants were transcribed with T7 RNA polymerase from PCR products obtained with primers described on Table III and relevant plasmid eluted from agarose gel as DNA templates. After deposphorylation with Calf intestine alkaline phosphatase (Fermentas), RNA was 5′-end labelled with [γ-^32^P]ATP and T4 polynucleotide kinase (Fermentas). Labelled RNAs were then purified by 10% polyacrylamide/7M urea/1x TBE gel electrophoresis, eluted and precipitated with ethanol.

### Chemical Probing

The dimethyl sulphate (DMS) modification of unpaired adenosine and cytidine nucleotides was carried out essentially as described [59]. For *in vitro* reactions, a 25 µl of total RNA renatured in Na-Cacodylate/EDTA buffer supplemented with 10 mM MgCl_2_ was treated with 1 µl freshly prepared DMS (Sigma-Aldrich) solution (diluted 1∶7 in ethanol) for 10 minutes at 37°C. Reaction was stopped with addition of 475 µl of quenching solution (4.3 M β-mercaptoethanol/0.3 M sodium acetate) and RNAs were precipitated overnight at −80°C. The *in vivo* DMS modification of RNA from stationary phase cultures was performed essentially as described [60], [61]. Primer extension reactions were carried out using a [γ-^32^P]-5′-end labelled MicA-DMS LNA™ primer (Exiqon) and the Transcriptor reverse transcriptase (Roche). After RNA alkaline hydrolysis, cDNA was resuspended in 6 µl formamide loading buffer. Samples were analysed on 6% or 8% polyacrylamide/7M urea gels run in TBE 1x buffer. The lead acetate cleavages were carried out as decribed [62] with addition of 5 mM PbAc (Sigma-Aldrich) to renatured 5′-end-labelled MicA RNAs in structure buffer 1x (Ambion) supplemented with 0.1 mg/ml of yeast RNA. Samples were collected after incubation for 0.5, 1 or 2 minutes and reactions were stopped by addition of 10 µl of loading buffer II (Ambion). In line probing was performed as described [63]. Samples were fractionated on 10% polyacrylamide/7M urea gels run in TBE 1x buffer. Gels were dried and exposed on the phosphor screen.

### Enzymatic Probing

Ribonucleases T1 (0.01 U) and RNase A (0.01 U) (Ambion) were incubated with the RNA for 15 minutes at 37°C following manufacturer’s instructions. Before use, 5′-end labelled RNAs were renatured in structure buffer 1x (Ambion). Unfolded RNAs were prepared in sequencing buffer 1x and a ladder of G-specific cleavages was obtained upon RNase T1 incubation. Alkaline ladders correspond to incubation of the RNA in the alkaline hydrolysis buffer for 15 minutes at 90°C. Reactions were stopped by adding 10 µl of loading buffer II (Ambion). Samples were then fractionated on 10% polyacrylamide/7 M urea gels run in TBE 1x buffer. Gels were dried and exposed on the phosphor screen.

### RNase III Cleavage Assay of MicA RNA

Reactions were performed using 1000 µM RNase III purified protein (kindly provided by Allen Nicholson) and radiolabeled [α-^32^P]-UTP MicA-WT RNA as substrate. RNase III reaction buffer consisted of 160 mM NaCl, 30 mM Tris–HCl (pH 8), 0.1 mM EDTA, 0.1 mM DTT and 10 mM MgCl_2_ [64]. Addition of the enzyme started the reaction and samples were collected at different timepoints. Incubation was performed at 37°C. Reactions were stopped by the addition of formamide loading buffer supplemented with 20251658240mM EDTA. Reaction products were resolved in a 15% polyacrylamide/7251658240M urea gel. Signals were visualized by PhosphorImaging and analysed using the ImageQuant software (Molecular Dynamics).

## Supporting Information

Figure S1 **Identification of the nucleotide changes introduced in the synthetic MicA variants.** *E. coli* MicA wild-type sequence is indicated on top and the mutated MicA variants are shown below. Designation of each MicA variant is indicated on the left of each sequence. A multiple alignment of MicA in several eubacteria (see Figure 1) identified the conserved nucleotides (*) in MicA sequence. A color-code was used to better scheme the domains of MicA: the 5′ linear domain (blue), the stem-loop 1 (red), the Hfq-binding site A/U-rich sequence (green), the stem-loop 2 (brown) and the 3′ poly(U) terminator tail (purple). Mutated nucleotides are shown in lowercase; if conserved, the residue is also underlined.
(TIF)

Figure S2 **In line probing analysis of MicA RNAs.** 5′-end labeled MicA-RNA was prepared in 50 mM Tris pH8, 20 mM MgCl2 and 100 mM KCl. In line probing reactions [69] were carried out for 48 h at room temperature and were stopped with addition of loading buffer II (Ambion). Untreated controls (C1: MicA-5′mut; C2: MicA-WT; C3: MicA-STEM1_2). Alkaline ladders and RNase T1 ladders were run on the same gel (data not shown). Thick lines on the side of the lanes represent the position of stem-loop arms. Samples were fractionated on 10% polyacrylamide/7M urea gels run in TBE 1x buffer.
(TIF)

Figure S3 ***In vitro*** **RNase III cleavage assay.** *In vitro* activity assay [70] with 1000 µM purified RNase III and radioactive labelled wild-type MicA RNA as substrate. Addition of RNase III started the reaction and samples were taken across time. A parallel reaction without the addition of enzyme was used as control. A size marker is shown on the left of the gel.
(TIF)

Figure S4 **Predicted Interactions between MicA-WT and the synthetic MicA variants with** ***omp*** **A,** ***tsx*** **and** ***ecn*** **B mRNAs.** The RNAhybrid software [71] was used to predict interactions between MicA forms and target mRNAs (*omp*A mRNA, *tsx* mRNA and *ecn*B mRNA), using the default parameters. A segment of the 5′ end of each target mRNA was chosen as previously described [72]. The complete sequences of all MicA variants were used. Nucleotide changes are shown underlined. For representative purposes, the predicted domains of MicA are color-coded: the 5′ linear domain (blue), the stem-loop 1 (red), the Hfq-binding site A/U-rich sequence (green), the stem-loop 2 (brown) and the 3′ poly(U) terminator tail (purple).
(TIF)

Figure S5 **Mutagenesis of the 3′ end U-rich tail of RybB.** Decay measurement of the RybB. Deleted *ryb*B cells (Δ*ryb*B) were transformed with a plasmid expressing either the wild-type copy (pRybB-WT) or a RybB variant in which the 3′ U-rich tail was modified to a CG-rich sequence (pRybB-3′mut). Total RNA was extracted from stationary phase cultures.
(TIF)

Figure S6 **Northern blot analysis of MicA RNA.** A band denoted nonspecific is detected on Northern blot analysis from RNA extracted from the wild-type (wt) and Δ*mic*A strains (transformed or not with plasmid pMicA-WT) when using the MicA riboprobe described in *Materials and Methods.*
(TIF)
