# Correction: Small RNA Modules Confer Different Stabilities and Interact Differently with Multiple Targets

**DOI:** 10.1371/journal.pone.0102277

**Published:** 2014-07-02

**Authors:** The PLOS ONE Staff

In the Funding section, the grant number from the funder Fundação para a Ciência e a Tecnologia (FCT) is missing. The correct grant number is: PTDC/CVT/102293/2008.
