# Supplementary figures and images for: Small RNA Modules Confer Different Stabilities and Interact Differently with Multiple Targets
Source: PLoS One. 2013 Jan 22;8(1):e52866. doi: 10.1371/journal.pone.0052866 (PMC3551931; doi:10.1371/journal.pone.0052866)

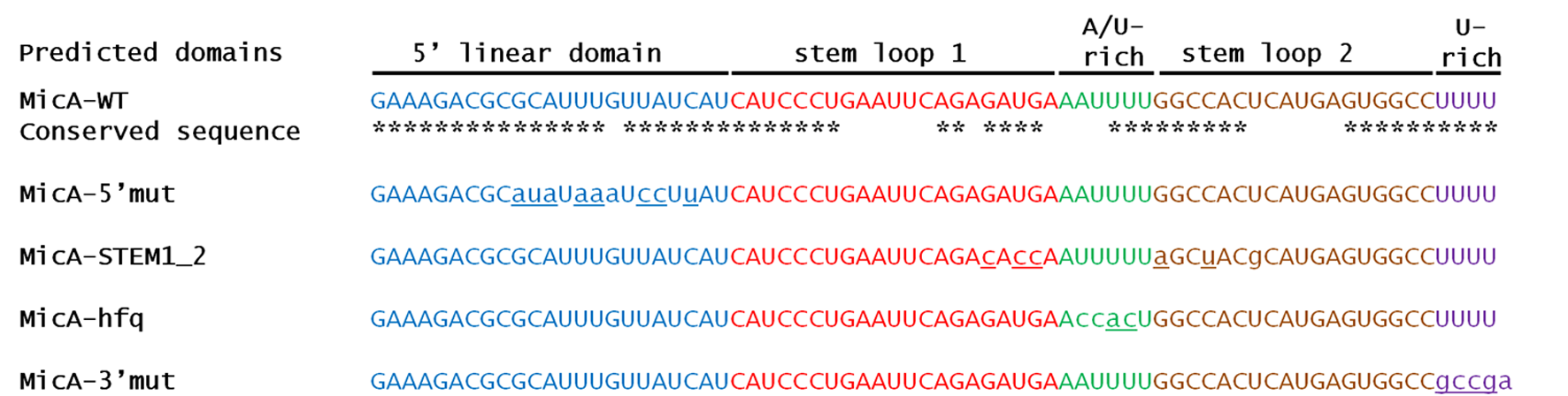

Supplement: Figure S1 — Identification of the nucleotide changes introduced in the synthetic MicA variants. E. coli MicA wild-type sequence is indicated on top and the mutated MicA variants are shown below. Designation of each MicA variant is indicated on the left of each sequence. A multiple alignment of MicA in several eubacteria (see Figure 1) identified the conserved nucleotides (*) in MicA sequence. A color-code was used to better scheme the domains of MicA: the 5′ linear domain (blue), the stem-loop 1 (red), the Hfq-binding site A/U-rich sequence (green), the stem-loop 2 (brown) and the 3′ poly(U) terminator tail (purple). Mutated nucleotides are shown in lowercase; if conserved, the residue is also underlined. (TIF) [file pone.0052866.s001.tif]

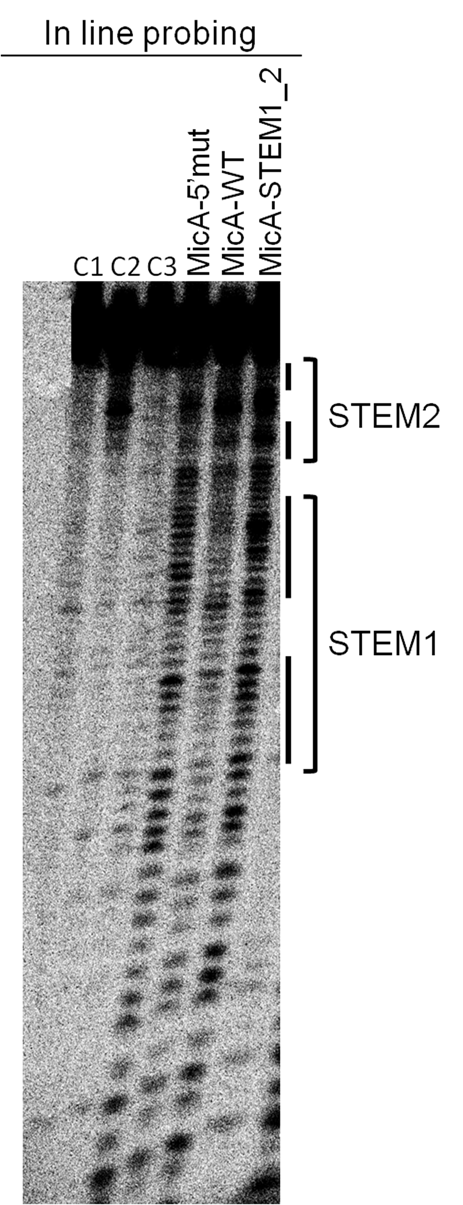

Supplement: Figure S2 — In line probing analysis of MicA RNAs. 5′-end labeled MicA-RNA was prepared in 50 mM Tris pH8, 20 mM MgCl2 and 100 mM KCl. In line probing reactions [69] were carried out for 48 h at room temperature and were stopped with addition of loading buffer II (Ambion). Untreated controls (C1: MicA-5′mut; C2: MicA-WT; C3: MicA-STEM1_2). Alkaline ladders and RNase T1 ladders were run on the same gel (data not shown). Thick lines on the side of the lanes represent the position of stem-loop arms. Samples were fractionated on 10% polyacrylamide/7M urea gels run in TBE 1x buffer. (TIF) [file pone.0052866.s002.tif]

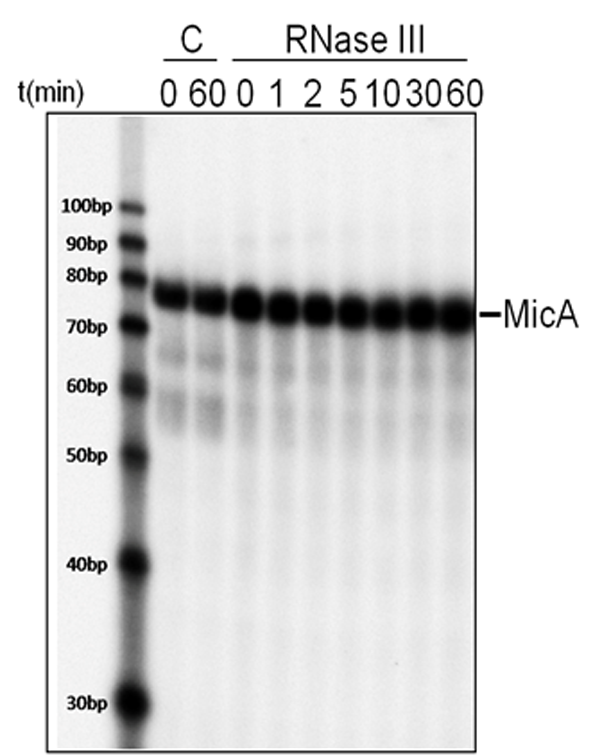

Supplement: Figure S3 — In vitro RNase III cleavage assay. In vitro activity assay [70] with 1000 µM purified RNase III and radioactive labelled wild-type MicA RNA as substrate. Addition of RNase III started the reaction and samples were taken across time. A parallel reaction without the addition of enzyme was used as control. A size marker is shown on the left of the gel. (TIF) [file pone.0052866.s003.tif]

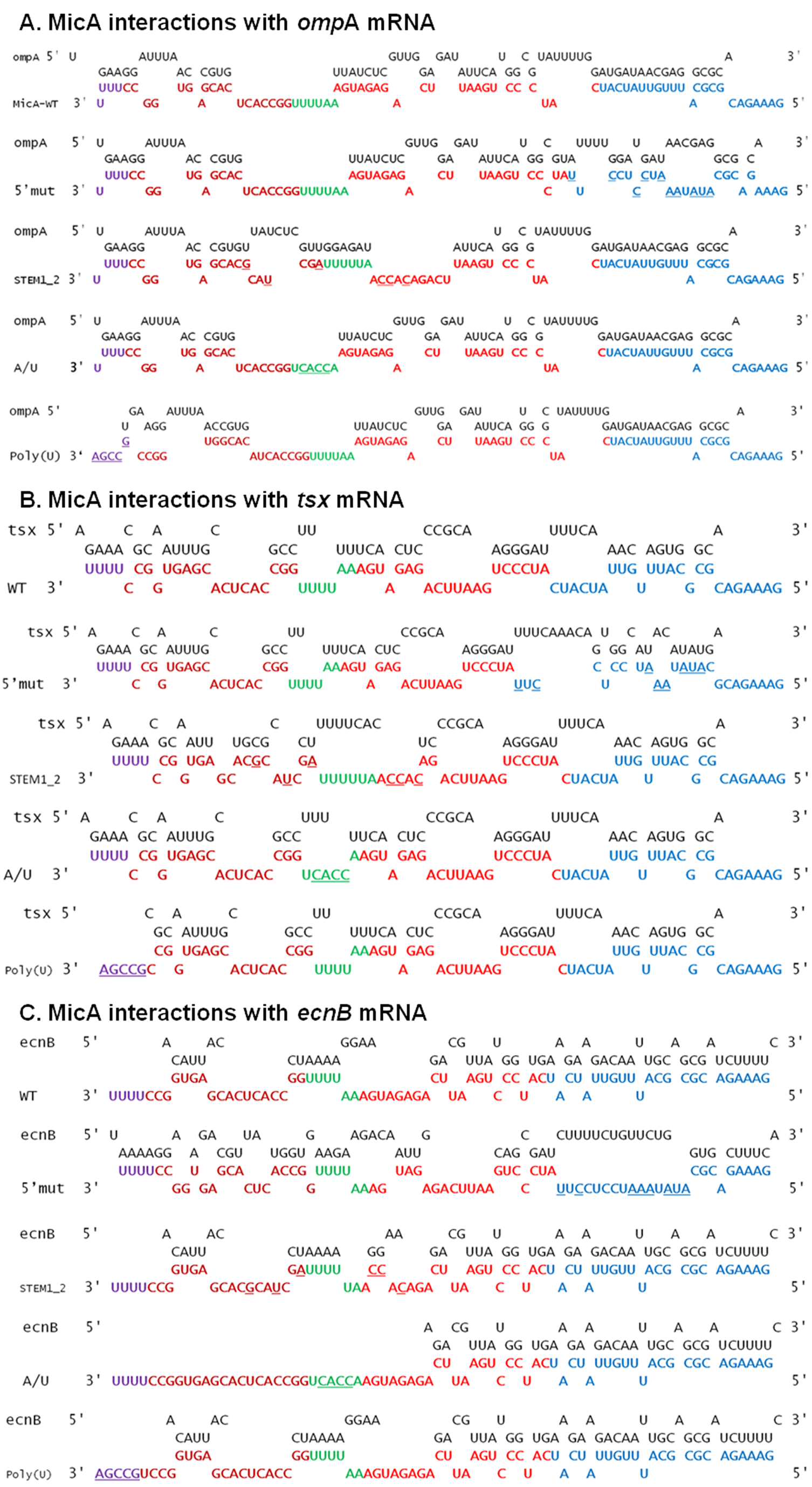

Supplement: Figure S4 — Predicted Interactions between MicA-WT and the synthetic MicA variants with omp A, tsx and ecn B mRNAs. The RNAhybrid software [71] was used to predict interactions between MicA forms and target mRNAs (ompA mRNA, tsx mRNA and ecnB mRNA), using the default parameters. A segment of the 5′ end of each target mRNA was chosen as previously described [72]. The complete sequences of all MicA variants were used. Nucleotide changes are shown underlined. For representative purposes, the predicted domains of MicA are color-coded: the 5′ linear domain (blue), the stem-loop 1 (red), the Hfq-binding site A/U-rich sequence (green), the stem-loop 2 (brown) and the 3′ poly(U) terminator tail (purple). (TIF) [file pone.0052866.s004.tif]

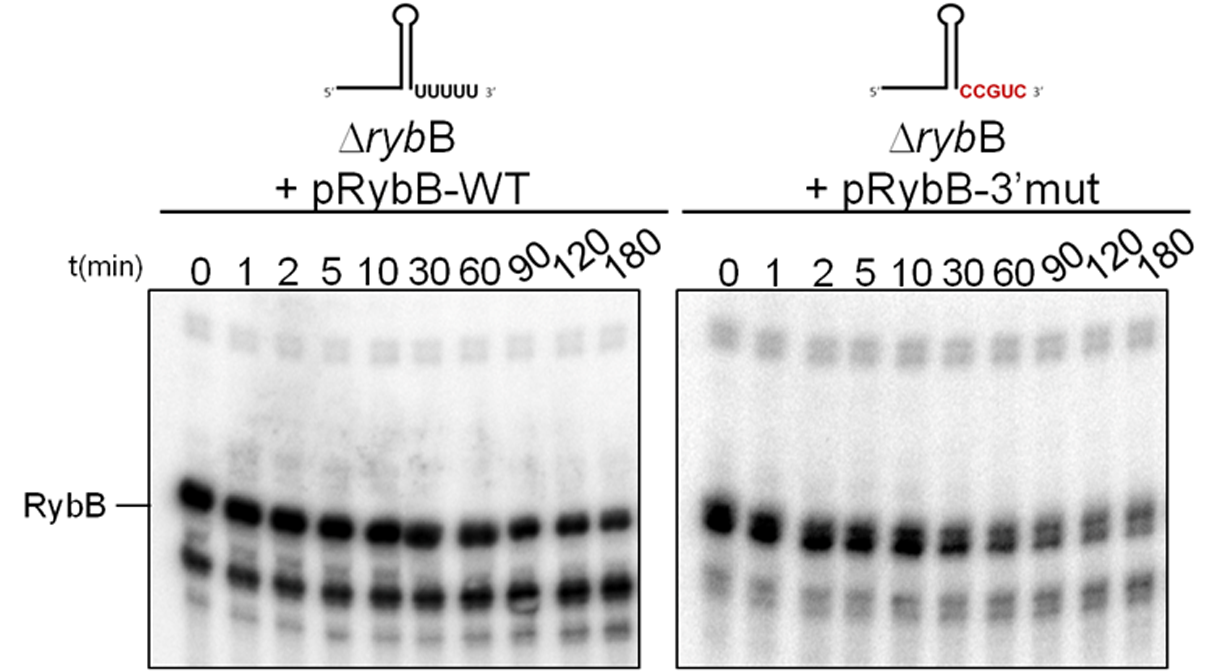

Supplement: Figure S5 — Mutagenesis of the 3′ end U-rich tail of RybB. Decay measurement of the RybB. Deleted rybB cells (ΔrybB) were transformed with a plasmid expressing either the wild-type copy (pRybB-WT) or a RybB variant in which the 3′ U-rich tail was modified to a CG-rich sequence (pRybB-3′mut). Total RNA was extracted from stationary phase cultures. (TIF) [file pone.0052866.s005.tif]

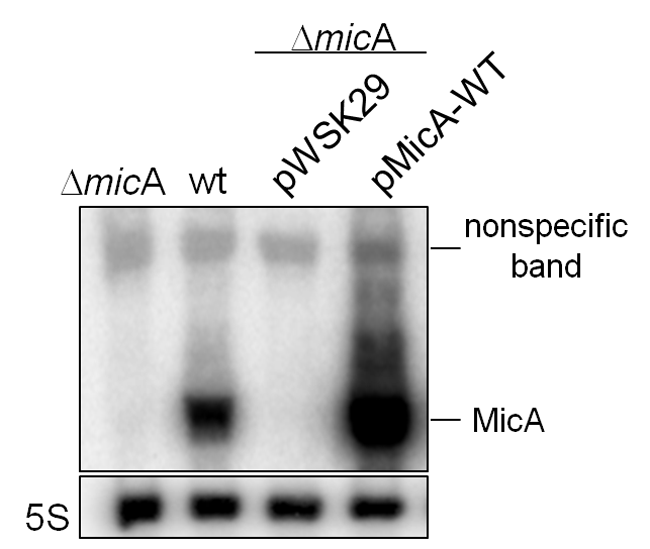

Supplement: Figure S6 — Northern blot analysis of MicA RNA. A band denoted nonspecific is detected on Northern blot analysis from RNA extracted from the wild-type (wt) and ΔmicA strains (transformed or not with plasmid pMicA-WT) when using the MicA riboprobe described in Materials and Methods. (TIF) [file pone.0052866.s006.tif]
